# Supplementary material for: Prevalence and Motivators of Getting a COVID-19 Booster Vaccine in Canada: Results from the iCARE Study
Source: Vaccines (Basel). 2023 Jan 28;11(2):291. doi: 10.3390/vaccines11020291 (PMC9960725; doi:10.3390/vaccines11020291)
Supplement: Supplementary file 1 [file vaccines-11-00291-s001.zip › vaccines-2158666-supplementary.pdf]

Supplementary Table S1. Participant characteristics of the overall sample

	Survey 9<br>(Jan. 21 -Feb 4, 2022)<br>(N=3001)<br>% (N)
**Sex**	
Male	48.4 (1446)
Female	51.6 (1543)
Missing values	12
**Age**	
Less than or equal to 25 years	12.5 (371)
26-50 years	40.3 (1197)
51 years or more	47.2 (1402)
Missing values	31
**Education level**	
High school diploma and less	72.6 (2159)
Cegep and more	27.4 (816)
Missing values	26
**Income**	
Less than 60K	46.8 (1251)
60K and more	45.2 (1423)
Missing values	327
**Chronic Disease**	
No	54.8 (1593)
At least one chronic disease	45.2 (1316)
Missing values	92
**Depressive Disorder**	
Yes	18.4 (539)
No	81.6 (23880
Missing values	74
**Anxiety Disorder**	
Yes	23.0 (671)
No	77.0 (2250)
Missing values	80
**Parent**	
No	78.8 (2304)
Yes	21.2 (621)
Missing values	76
**Healthcare Worker**	
No	95.3 (2783)
Yes	4.7 (136)

Missing values	82
**Essential Worker**	
No	80.1 (2344)
Yes	19.9 (582)
Missing values	75
**Infected with COVID-19**	
Yes	78.4 (2167)
No	21.6 (598)
Missing values	236
**Received a COVID-19 vaccine booster dose**	
Yes	67.0 (1744)
No	33.0 (859)
Missing values	398

Supplementary Table S2. Frequencies of motivators «to a great extent» as a function of booster vaccination status

Motivators	Not boosted (N=859) % (N)	Boosted (N=1744) % (N)
Trusting the company who developed the booster	32.3 (259)	44.1 (752)
Having information that the booster is safe and unlikely to have any serious short-term side effects	41.6 (337)	55.7 (957)
Having information that the booster is safe and unlikely to have any serious long-term side effects	44.2 (360)	56.6 (972)
Having information that the booster is effective	44.8 (365)	64.8 (1118)
Believing that I am high risk of getting infected with COVID-19	25.4 (197)	30.3 (502)
Hearing that other people have positive attitudes towards the booster	20.3 (163)	30.4 (516)
Seeing more people etting the booster	18.9 (153)	29.4 (502)
Learning that getting the ooster would allow me to go to restaurants/bars, attend public events or travel	27.1 (214)	39.9 (657)
Wanting to do my part to achieve "herd immunity"	35.5 (283)	64.8 (1114)
Getting a recommendation fro my employer to get the booster	17.7 (112)	20.7 (202)
Getting the booster according to the recommended schedule	25.9 (200)	52.2 (888)
Believing that getting the booster would reduce my worries and anxiety	21.9 (172)	42.9 (725)
Getting a recommendation from my doctor to get the booster	21.5 (155)	31.9 (425)
The convenience of getting the booster	28.3 (224)	44.5 (755)
Knowing that getting the booster will help protect others around me	39.2 (319)	69.7 (1198)
Having information that the booster is effective against new COVID-19 straints or variants	41.0 (332)	60.7 (1038)
Having a choice about which booster I get	35.3 (279)	31.3 (508)
Getting clear and consistent booster information from my government	34.9 (279)	47.2 (800)

Supplementary Table S3. Participants motivators as a function of booster vaccination status

Motivators	Boosted (Reference : not boosted)			
	OR	95% CI Lower	95% CI Upper	p-value
Trusting the company who developed the booster	1.17	0.93	1.46	0.183
Having information that the booster is safe and unlikely to have any serious short-term side effects	**1.40**	1.13	1.73	**0.002**
Having information that the booster is safe and unlikely to have any serious long-term side effects	**1.35**	1.09	1.67	**0.007**
Having information that the booster is effective	**1.78**	1.43	2.21	**<.001**
Believing that I am high risk of getting infected with COVID-19	1.12	0.87	1.45	0.372
Hearing that other people have positive attitudes towards the booster	**1.55**	1.20	2.00	**0.001**
Seeing more people getting the booster	**1.65**	1.28	2.14	**0.001**
Learning that getting the booster would allow me to go to restaurants/bars, attend public events or travel	**1.55**	1.23	1.94	**0.001**
Wanting to do my part to achieve "herd immunity"	**3.27**	2.62	4.07	**<.001**
Getting a recommendation from my employer to get the booster	0.94	0.69	1.28	0.708
Getting the booster according to the recommended schedule	**2.53**	2.00	3.20	**<.001**
Believing that getting the booster would reduce my worries and anxiety	**2.08**	1.63	2.65	**<.001**
Getting a recommendation from my doctor to get the booster	**1.59**	1.22	2.07	**0.001**
The convenience of getting the booster	**1.66**	1.33	2.08	**<.001**
Knowing that getting the booster will help protect others around me	**3.11**	2.50	3.87	**<.001**
Having information that the booster is effective against new COVID-19 straints or variants	**1.80**	1.45	2.24	**<.001**
Having a choice about which booster I get	**0.67**	0.53	0.84	**0.001**

Getting clear and consistent booster information from my government	**1.40**	1.13	1.74	**0.002**

OR: Odds ratio, CI: Confidence Interval
Adjusted for: sex, age, education, income, having a chronic disease or mental disorder, being parent, being a healthcare or essential workers, COVID-19 infection, and weighting
